# Molecular Epidemiology and Risk Factors of *Clostridium difficile* ST81 Infection in a Teaching Hospital in Eastern China

**DOI:** 10.3389/fcimb.2020.578098

**Published:** 2020-12-23

**Authors:** Ziyu Yang, Qian Huang, Juanxiu Qin, Xiaoye Zhang, Ying Jian, Huiying Lv, Qian Liu, Min Li

**Affiliations:** ^1^Department of Laboratory Medicine, Renji Hospital, School of Medicine, Shanghai Jiaotong University, Shanghai, China; ^2^The Second Affiliated Hospital of Shandong University of Traditional Chinese Medicine, Shandong, China

**Keywords:** *Clostridium difficile* infection, multilocus sequence typing, epidemiology, risk factors, resistance

## Abstract

**Background:**

The prevalence of *Clostridium difficile* causes an increased morbidity and mortality of inpatients, especially in Europe and North America, while data on *C. difficile* infection (CDI) are limited in China.

**Methods:**

From September 2014 to August 2019, 562 *C. difficile* isolates were collected from patients and screened for toxin genes. Multilocus sequence typing (MLST) and antimicrobial susceptibility tests by E-test and agar dilution method were performed. A case group composed of patients infected with sequence type (ST) 81 *C. difficile* was compared to the non-ST81 infection group and non CDI diarrhea patients for risk factor and outcome analyses.

**Results:**

The incidence of inpatients with CDI was 7.06 cases per 10,000 patient-days. Of the 562 C*. difficile* isolates, ST81(22.78%) was the predominant clone over this period, followed by ST54 (11.21%), ST3 (9.61%), and ST2 (8.72%). Toxin genotype *tcdA+tcdB+cdt-* accounted for 50.18% of all strains, while 29.54% were *tcdA-tcdB+cdt-* genotypes. Overall, no isolate was resistant to vancomycin, teicoplanin or daptomycin, and resistance rates to meropenem gradually decreased during these years. Although several metronidazole-resistant strains were isolated in this study, the MIC values decreased during this period. Resistance rates to moxifloxacin and clindamycin remained higher than those to the other antibiotics. Among CDI inpatients, longer hospitalization, usage of prednisolone, suffering from chronic kidney disease or connective tissue diseases and admission to emergency ward 2 or emergency ICU were significant risk factors for ST81 clone infection. All-cause mortality of these CDI patients was 4.92%(n=18), while the recurrent cases accounted for 5.74%(n=21). The 60-day mortality of ST81-CDI was significantly higher than non-ST81 infected group, while ST81 also accounted for most of the recurrent CDI cases.

**Conclusion:**

This study revealed the molecular epidemiology and risk factors for the dominant *C. difficile* ST81 genotype infection in eastern China. Continuous and stringent surveillance on the emerging ST81 genotype needs to be initiated.

## Introduction

*Clostridium difficile* is a Gram-positive, spore-forming anaerobic bacillus that is a major cause of healthcare-associated infection known as *C. difficile* infection (CDI) (Jin et al., 2017; Czepiel et al., 2019; Guh et al., 2020). CDI is one of the principal threats to hospitalized and immunocompromised patients, with clinical manifestations ranging from asymptomatic carriers or mild diarrhea to fulminant infectious colitis occasionally complicated by toxic megacolon, sepsis, and death (Rupnik et al., 2009; Brkic et al., 2016). Recently, the incidence and mortality associated with CDI has increased dramatically, making *C. difficile* one of the most formidable emerging pathogens of our time (Rupnik et al., 2009; Jones et al., 2013). The CDI-attributable mortality ranges from 6.9% to 16.7% in epidemic periods as reported previously (Kwon et al., 2015). Toxin A and B (TcdA and TcdB) are the major virulence factors of *C. difficile* (Kuehne et al., 2010). However, some strains can also produce a third unrelated binary toxin (CDT) encoded by *cdtA* and *cdtB* genes (Persson et al., 2011).

For the past 30 years, metronidazole and vancomycin have remained the first-line agents for the treatment of CDI (Debast et al., 2014). However, several clinical studies have reported reduced susceptibility or resistance of *C. difficile* to both antibiotics (Peláez et al., 2008; Freeman et al., 2015). Therefore, knowledge of the antimicrobial susceptibility profiles of *C. difficile* is not only an important first step for understanding the epidemiology of this organism, but also offers information about the persistence of specific types over time in hospital settings.

Multilocus sequence typing (MLST) analysis, based on allelic polymorphisms in 7 housekeeping genes, has commonly been used to analyze evolutionary genetic changes in *C. difficile* (Lemée and Pons, 2010). The genotypes of *C. difficile* have varied in different populations and regions. While ST1 has been the dominant clone for years in North America and Europe (Thorpe et al., 2019; Piepenbrock et al., 2019), Tang et al. demonstrated that the most predominant sequence type in Shanghai used to be ST37, followed by ST35 and ST3, while in Beijing it was ST35, followed by ST3 and ST54 (Tang et al., 2016). Genetic diversity of *C. difficile* isolates over the years within a region is dynamically changing, and thus continuous monitoring of *C. difficile* is essential for surveillance.

Our study comprehensively described 562 C*. difficile* clinical isolates collected in a tertiary teaching hospital in Shanghai, China, from September 2014 to August 2019. The aim of this study was to investigate molecular genotypes, antimicrobial resistance patterns, presumed risk factors as well as clinical outcomes of the dominant sequence type in a large scale to fill the void of clinical epidemiological data on *C. difficile* in Eastern China.

## Methods

### Study Design and Bacterial Isolates

This was a retrospective study to survey the molecular epidemiology and risk factors associated with CDI. From 2014 to 2019, a total of 668 C*. difficile* isolates were collected in a comprehensive teaching hospital in Shanghai, China (Renji Hospital, affiliated with Jiaotong University) and maintained in MICROBANK (PRO-LAB, Canada) at -80°C for long-term storage. However, only 562 isolates were successfully recovered on the *C. difficile* selective medium CDIF (BioMerieux, France) and verified by Matrix-Assisted Laser Desorption/Ionization Time of Flight Mass Spectrometry (MALDI-TOF MS, Bruker, German). All the patients included in the study were diagnosed with CDI by the following criteria: the presence of consecutive and unformed stools tested positive for the presence of toxigenic *C. difficile* or its toxins, or histopathologic or colonoscopic findings indicating pseudomembranous colitis (Cohen et al., 2010). This study was approved by the ethics committee of Renji Hospital, School of Medicine, Shanghai Jiaotong University, Shanghai, China(Ethical number: KY2020-108).

### Definitions and Data Collection

*C. difficile* isolates resistant to three classes of antibiotics were defined as multidrug resistance (MDR). ST81-infected group was compared to two control groups. One was non-ST81-infected group, and the other was composed of the randomly selected diarrheic inpatients negative for *C. difficile*, matching (1:1) to ST81 CDI cases on admission time. Patients’ information was extracted from patients’ electronic medical records and comprehensively reviewed. The variables applied in the molecular epidemiology and risk factor analysis include: 1) demographics (gender, age) of both inpatients and outpatients; 2) therapeutic process during hospital stay (length of hospitalization, medical history before and during hospitalization (antibiotics, prednisolone, immune inhibitors, and proton pump inhibitors), department and ward, enteroscopy, and blood transfusion); 3) underlying diseases (hypertension, diabetes mellitus, chronic kidney diseases, chronic liver diseases, cardio-cerebrovascular diseases, peptic ulcer, benign or malignant tumors, connective tissue diseases, and severe infection including sepsis, systemic inflammatory response syndrome and septic shock) and comorbidities (evaluated by Charlson index, an applicable and valid method of predicting 10-year survival in patients with multiple comorbidities) (Charlson et al., 1987). Chronic kidney disease(CKD) is defined as kidney damage or a GFR<60 ml/min/1.73 m^2^ for a period longer than 3 months (Drawz and Rahman, 2015). Patients with the following diagnosis were classified into chronic liver disease (CLD) group: liver transplant, cirrhosis, fatty liver disease, HBV, HCV, and autoimmune liver disease (Rustgi et al., 2020). The 60-day mortality is defined as an all-cause death ratio within 60 days since the diagnosis of CDI. Recurrent CDI (rCDI) refers to a new episode occurring within 8 weeks of a previous successfully treated episode (Lei et al., 2019).

### Multi-Locus Sequence Typing

MLST was performed and analyzed as previously described (Griffiths et al., 2010). Briefly, seven loci (*adk, atpA, dxr, glyA, recA, sodA*, and *tpi*) were amplified by PCR and sequenced, of which the results were submitted to the public MLST database (https://pubmlst.org/cdifficile/). The online website PHYLOViZ (http://online.phyloviz.net/index) was used to analyze the evolutionary relatedness of different sequence types based on the goeBURST algorithm (Francisco et al., 2009).

### Detection of *Clostridium difficile* Toxin Genes

Bacterial genomic DNA was extracted for PCR. The housekeeping gene *tpi*, toxin genes *tcdA* (toxin A) and *tcdB* (toxin B), and binary toxin genes *cdtA* and *cdtB* were detected with primer sequences as previously described (Persson et al., 2008; Griffiths et al., 2010). All the primers used for molecular typing can be found in **Supplementary Table 1**.

### Antimicrobial Susceptibility Testing

Antimicrobial susceptibility to the routinely applied broad-spectrum antibiotics against bacterial infection, as well as to those effective in CDI treatment was tested as previously reported (Qin et al., 2017; Tickler et al., 2019; Aliramezani et al., 2019). The susceptibility of *C. difficile* isolates to six antimicrobial agents, including vancomycin, meropenem, linezolid, moxifloxacin, metronidazole, and teicoplanin were tested by E-test. Minimal inhibitory concentration (MIC) values of three antibiotics, rifaximin, daptomycin and clindamycin, were determined by agar dilution method according to Clinical and Laboratory Standards Institute (CLSI) guidelines. The *C. difficile* colonies recovered on 5% Columbia blood agar (Oxoid, UK) were then picked to make a 0.5 McFarland standard turbidity. Part of the inoculation was performed with cotton-tipped swabs and E-test strips were applied to the surface of Enhanced Brucella Broth(BBL, BD) solid medium supplemented with 5 mg/L hemin, 1 mg/L vitamin K1, and 5% defibrinated sheep red blood cells according to the manufacturer’s instructions, while a multipoint inoculator was used in agar dilution method. The mediums were incubated at 37°C for 48 h in an anaerobic chamber and MIC values were recorded. ATCC 70057 is a standard *C. difficile* strain, and was used for quality control. Interpretation of the MIC results was based on the CLSI recommendations for metronidazole, moxifloxacin, clindamycin, meropenem, linezolid, and teicoplanin. The breakpoint of vancomycin was determined by the European Committee on Antimicrobial Susceptibility Testing (EUCAST) (>2 mg/L; clinical breakpoints bacteria v5.0; http://www.eucast.org/clinical_breakpoints/), while the breakpoint of rifaximin was according to that of rifampin. The breakpoint of daptomycin according to EUCAST guidelines was based on the epidemiological cut-off value for the “wild-type” population (>4 mg/L).

### Statistical Analysis

All statistical analyses were performed by SPSS 26.0 (IBM Corp., Armonk, NY, United States). Two-sided *p* values <0.05 were considered statistically significant. Continuous variables or medians were analyzed by nonparametric tests. χ^2^ or Fisher’s exact test was used to compare qualitative variables. Univariate analysis was used in risk factor evaluation for each variable. Variables with a *P* value of less than 0.05 were added in the multivariate logistic regression model to determine the potential independent risk factors associated with ST81 CDI. Odds ratios (ORs) and 95% confidence intervals (CIs) were calculated to evaluate the association between the factors and outcomes. Kaplan-Meier survival analysis was applied to compare mortality between the groups of ST81 CDI and non-ST81 CDI.

## Results

### Analysis of Clinical Data and Bacterial Isolates

**Figure 1:**
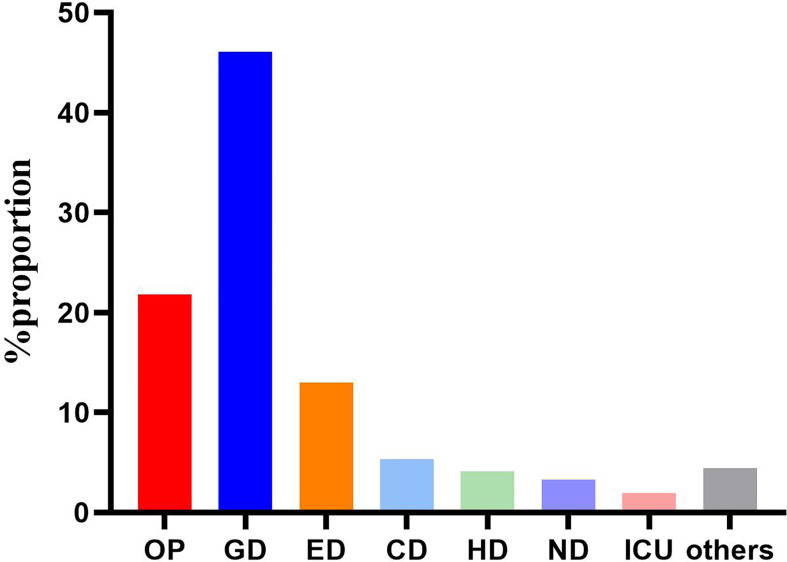
Distribution of the 562 strains according to admission departments. OP, Outpatients; GD, Gastroenterology department; ED, Emergency department; CD, cadre (geriatric) department; ND, Nephrology department; HD, Hematology department; ICU, Intensive care unit.

Totally, 562 *C. difficile* isolates were included in the study. Generally, 448 patients met the diagnostic criteria of *C. difficile* infection, including 336 inpatients and 112 outpatients. The clinical information of 336 inpatients infected by toxigenic *C. difficile* was collected to analyze the risk factors of ST81 CDI. Mean age of all the CDI patients was 54.06 ± 21.2 years, while 58.35% of them were male. Most of the patients (n=259, 46.09%) were from gastroenterology department, followed by emergency department (n=73, 12.99%), cadre (geriatric) department (n=30, 5.34%), hematology department (n=23, 4.09%), nephrology department (n=18, 3.26%), intensive care unit (n=11, 1.96%), and others accounting for the remaining 4.45% (**Figure 1**).

### Genotypes and Phylogenetic Analysis

**Figure 2:**
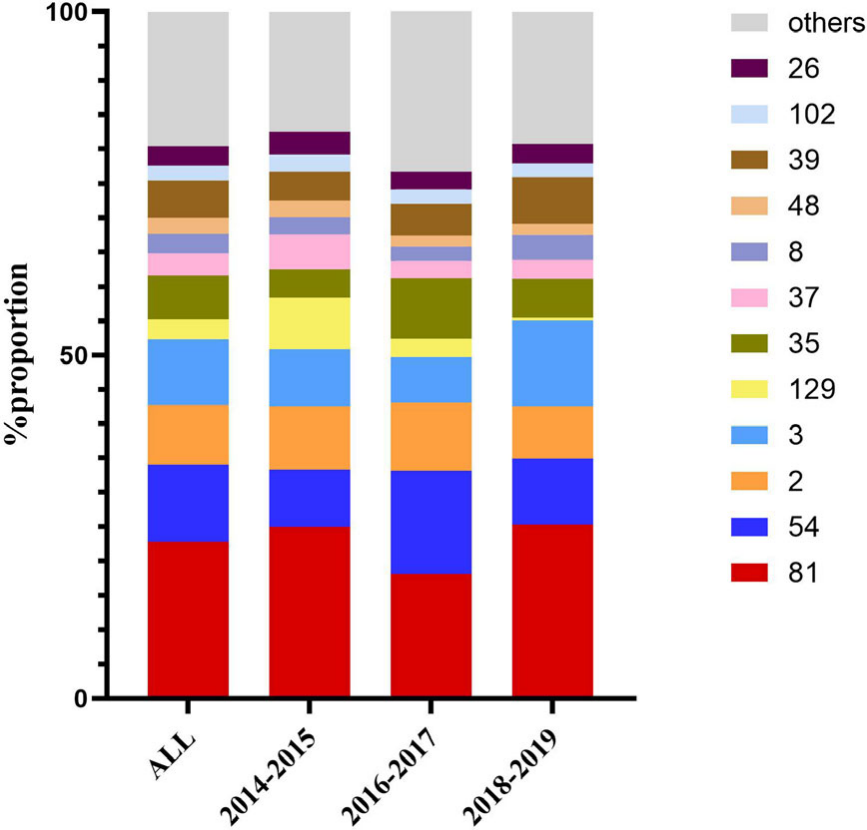
Dynamic changes in proportion of the most frequent sequence types over the three time periods.

**Figure 3:**
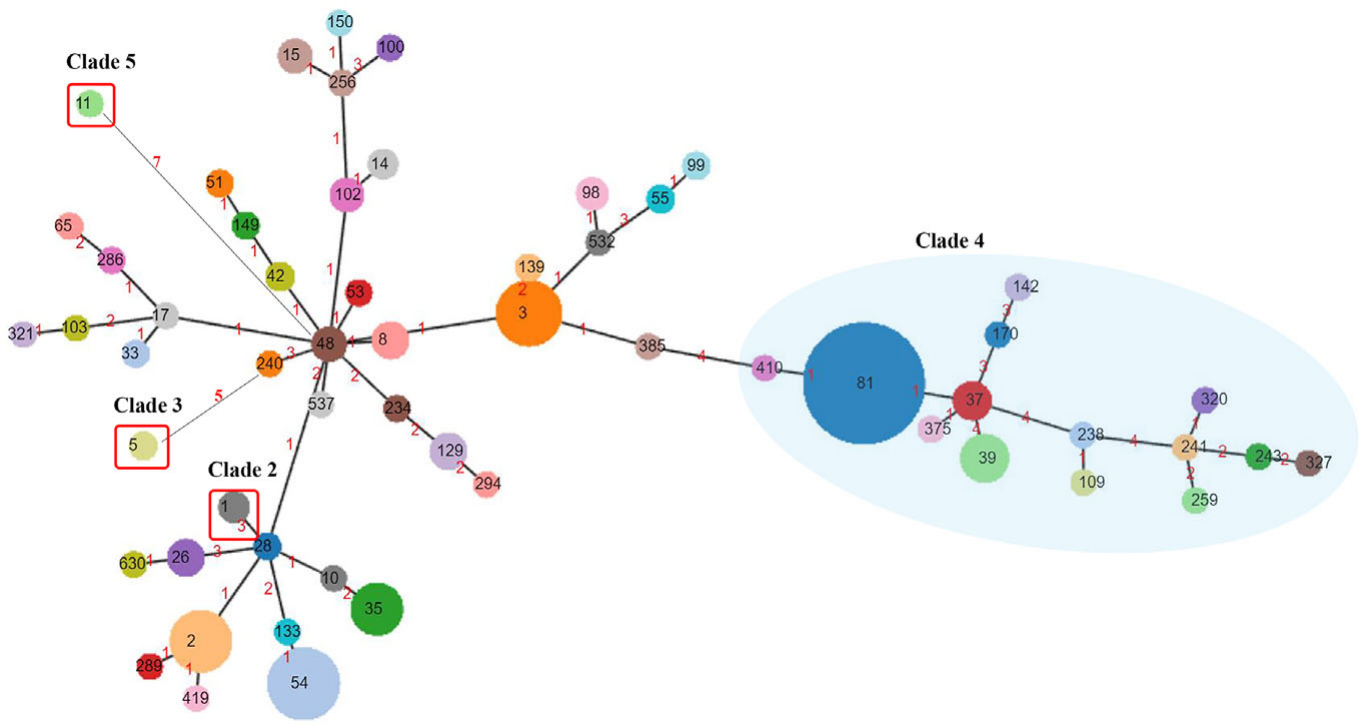
Phylogenetic analysis of *C. difficile*. ST numbers assigned are overprinted, while the node size is proportional to the quantity of isolates presenting in this database. Numbers of the links represent the amount of locus variants between the two STs. Clade 4 is clustered in a blue semitransparent circle. Clade 2, Clade 3, and Clade 5, each containing one ST genotype, are highlighted by red rectangular frames. The rest of ST genotypes all belong to Clade 1.

The 562 isolates were assigned to 57 STs during the 5 years, and ST81 remained the major ST of all, accounting for 22.78% (n=128). ST54 (n=63, 11.21%), ST3 (n=54, 9.61%), ST2 (n=49, 8.72%), ST35 (n=36, 6.41%), and ST39 (n=31, 5.52%) were also predominant genotypes. In contrast, as reported in other regions of China, the most prevalent STs were ST37 (Jin et al., 2017), ST2 (Luo et al., 2018), ST54 (Chen et al., 2018; Wang et al., 2018c), and ST35 (Liu et al., 2018). As shown in **Figure 2**, the proportions of ST39 and ST3 were increasing, while ST129 represented a decreasing trend (p=0.044). The phylogenetic relationships of the 562 isolates were analyzed by using an online website based on the ST patterns as shown in **Figure 3**. Five major clonal complexes (CC) were divided according to particular features related to ribotyping and toxin production (Knight et al., 2015). In line with a multi-center analysis, clade 1 (C1) was the most frequent (Muñoz et al., 2017) in this study (n=355, 63.17%), which appears to be highly heterogeneous, containing over 100 different STs, and many of them (such as ST54, ST2, ST3, and ST35) are epidemic and clinically significant. While ST37 was considered responsible for outbreaks in Europe and North America, and also prevalent in China (Li et al., 2019; Luo et al., 2019), ST81 has been found to be predominant over years in this hospital, which might be the reason why clade 4 has been the second most common CC (n=193, 34.34%) in this period. Notably, ST81 and ST37 are genetically related, and show resistance to clindamycin and fluoroquinolones clinically (Knight et al., 2015). Only one ST for each of clade 2, clade 3 and clade 5 has been detected in this study, which was ST1, ST5 and ST11 respectively, and all of them were *tcdA*+*tcdB*+*cdt*+.

**Figure 4:**
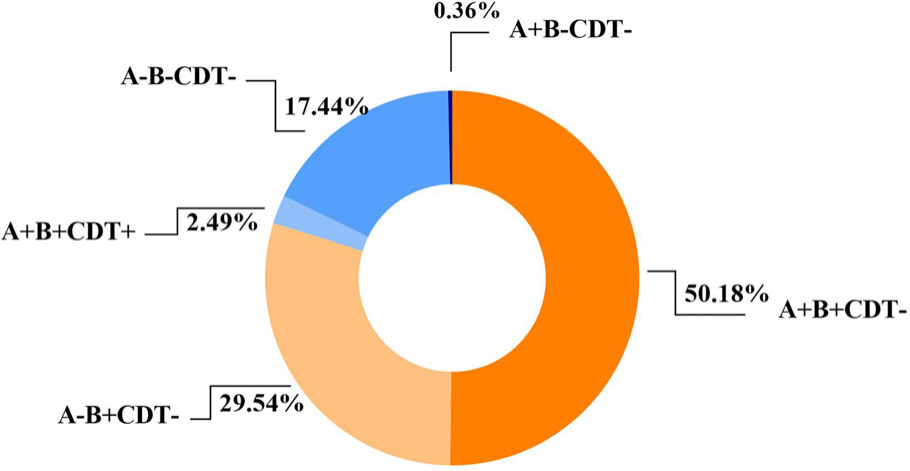
Proportion of toxin genotypes of the 562 strains.

All the toxin genes (*tcdA, tcdB, cdtA*, and *cdtB*) were screened. Of the 562 C*. difficile* isolates, 283 (50.18%) tested positive for both *tcdA* and *tcdB* genes, followed by 164 (29.54%) that were negative for *tcdA* and positive for *tcdB*, and 98 (17.44%) that were negative for both *tcdA* and *tcdB* (See **Figure 4**). Only 14 (2.49%) isolates possessing binary toxin genes (*cdtA* and *cdtB*), with *tcdA* and *tcdB* both positive, were isolated and considered as hypervirulent strains (Aktories et al., 2018). All ST81 and ST37 strains were positive exclusively for the *tcdB* gene, while ST54, ST35, and ST2 were positive for both *tcdA* and *tcdB* genes. ST3 isolates included both toxigenic (n=42, 77.78%) and non-toxigenic strains (n=12, 22.22%), while ST39, ST26, and ST48 were tested negative for all toxin genes.

### Correlations Between Major STs and Antimicrobial Resistance Patterns

**Figure 5:**
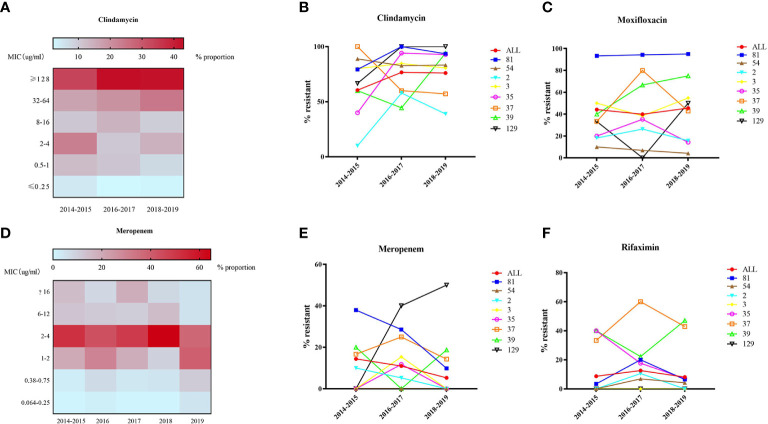
MIC distribution **(A, D)** and dynamic changes of resistance rate **(B, C, E, F)** to clindamycin, moxifloxacin, meropenem, and rifaximin of 562 C*. difficile* isolates.

All of the 562 isolates were enrolled for antimicrobial resistance tests. The antimicrobial resistance patterns for the nine antibiotics are listed in **Table 1**. No isolate resistant to vancomycin, teicoplanin and daptomycin was found with MIC_50_ values of 0.25, 0.125, and ≤0.064, respectively, while six isolates (1.07%) were resistant to metronidazole. As shown in **Figure 5A**, MIC of clindamycin represented a growing trend from 2014 to 2016 (p=0.007), and most of the strains showed high-level resistance (MIC≥32 mg/L) to clindamycin, with a MIC_50 =_ 64 mg/L and MIC_90 =_ 128 mg/L. ST81 has been highly resistant to clindamycin, with a resistance rate of 92.06% on average (**Figure 5B**). ST129 and ST35 showed continuous rising resistance rates to clindamycin, while the rate of ST37 was dramatically decreasing, which might be related to the decline of ST37 population. Generally, 43.39% of all the strains analyzed were resistant to moxifloxacin, among which the resistance rate of ST81 was significantly higher than the other STs and remained unchanged during these years (**Figure 5C**). In line with the MIC distribution of meropenem, a significant decline of resistance rate was also discovered in ST81, ST37, ST2, ST3 and ST35 (See **Figures 5D, E**). The ST37 clone was more resistant to rifaximin than the other STs (**Figure 5F**), however the resistance percentages of those major STs, including ST81, ST54, ST2, and ST37, were at their peak during 2016–2017 and have been declining since then. No isolate from ST2 and ST129 was resistant to rifaximin. Notably, the proportion of MDR ST39 was increasing, while MDR ST37 was disappearing in the most recent 2 years observed.

**Table 1:** MIC_50_, MIC_90_, and MIC range results for antimicrobial agents tested against 562 C*. difficile* isolates.

Antimicrobial agent^a^	All strains (n = 562)			
	MIC_50_ (mg/L)	MIC_90_ (mg/L)	MICrange (mg/L)	% Resistant
Meropenem (R ≥ 16)	2	8	0.064–≥32	9.19
Vancomycin (R ≥ 2)	0.25	0.38	0.016–1.5	**0**
Linezolid (R ≥ 4)	0.75	2	0.032–≥256	3.23
Metronidazole (R ≥ 32)	0.125	0.25	0.016–≥32	1.07
Moxifloxacin (R ≥ 8)	2	≥32	0.064–≥32	43.29
Teicoplanin (NA)	0.125	0.25	≤0.064–0.5	**0**
Rifaximin (R ≥ 4)	≤0.064	0.4	<0.064–>32	9.73
Daptomycin (NA)	≤0.064	≤0.064	≤0.064–>32	**0**
Clindamycin (R ≥ 8)	64	128	≤0.064–>128	73.06

a Resistance breakpoints (mg/L) in parentheses.

### Risk Factor Analysis for ST81 Clone Infection

**Figure 6:**
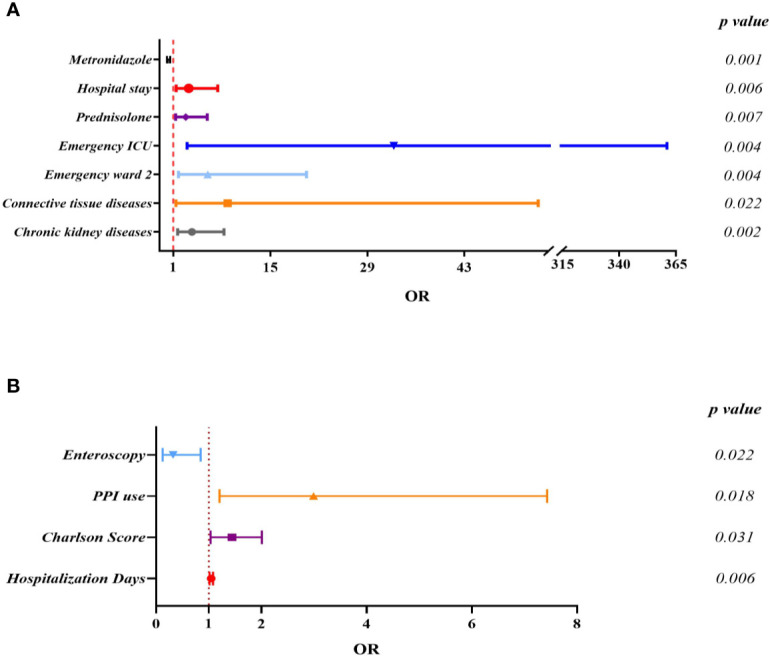
Multivariate logistic regression analysis for independent risk factors of ST81 CDI when non-ST81 CDI **(A)** or non-CDI diarrhea patients **(B)** were used as control.

Since ST81 was the most commonly isolated genotype in all these years with a high resistance rate to multiple clinically applied antimicrobial agents, the risk of ST81 infection should be thoroughly analyzed for better prevention and surveillance. Clinical information of 366 inpatients diagnosed with CDI has been collected and analyzed, among which 28.96% (n=106) were infected with ST81 *C. difficile*, and 71.04% (n=260) were characterized by non-ST81 CDI. Comparison of this case and control groups is listed in **Table 2**. Overall, the ST81 CDI group was significantly older than the non-ST81 CDI group, with a mean age of 64.73 ± 17.68 years versus 53.94 ± 22.10 years, respectively, while the gender ratio was similar in the two groups. In the univariate analysis, the ST81 CDI group was more likely to suffer from hypertension (OR: 3.183, p<0.001), diabetes mellitus (OR: 3.322, p<0.001), chronic kidney diseases (OR:5.462, p<0.001), chronic liver diseases (OR:2.451, p=0.001), cardiovascular and cerebrovascular diseases (OR:3.039, p<0.001), severe infection (OR:3.783, p<0.001), and connective tissue diseases (OR:5.140, p=0.022). Moreover, patients infected with ST81 *C. difficile* were burdened with more comorbidities (p<0.001) and stayed longer in hospital (p<0.001). Medication history was also included in the univariate analysis, where antibiotics used before and during hospitalization, usage of cephalosporins (4^th^ generation), carbapenems, fluoroquinolones, prednisolone and immune inhibitors were potential risk factors for ST81 CDI. According to the multivariable logistic regression model (see **Figure 6A**), longer days of hospitalization (OR: 3.232, CI: 1.410-7.409, p=0.006), admission in emergency ICU ward (OR: 32.803,CI: 2.980-361.084, p=0.004) and emergency ward 2 (OR: 5.954, CI: 1.753-20.223, p=0.004), along with prednisolone use (OR:2.795, CI: 1.331-5.871, p=0.007) were independent risk factors for ST81 CDI, while patients with chronic kidney diseases (OR:3.676, CI: 1.626-8.309, p=0.002) and connective tissue diseases (OR:8.833, CI: 1.378-53.667, p=0.022) were also characterized as high-risk population. Notably, the ST81 CDI group took less metronidazole during hospitalization (OR:0.196,CI: 0.072-0.535, p=0.001), though all ST81 strains isolated from these inpatients were susceptible to metronidazole.

**Table 2:** Characteristics of the ST81-infected and non-ST81 CDI groups.

Variables		ST81 CDI (n = 106)	Non-ST81 CDI (n = 260)	Univariate analysis	
		N (%)	N (%)	OR (95% CI)	p-value
**Demographics**					
Age (years, x ± SD)		64.73 ± 17.68	53.94 ± 22.10		**<0.001**
Gender: male		68(64.15)	161(61.92)	0.909(0.568–1.453)	0.690
**Diseases**					
Hypertension		41(38.68)	43(16.54)	3.183(1.912–5.300)	**<0.001**
Diabetes mellitus		22(20.75)	19(7.31)	3.322(1.713–6.441)	**<0.001**
Chronic kidney diseases		46(43.40)	32(12.31)	5.462(3.205–9.311)	**<0.001**
Chronic liver diseases		34(32.08)	42(16.15)	2.451(1.450–4.143)	**0.001**
Cardio-cerebrovascular diseases		47(44.34)	54(20.77)	3.039(1.868–4.943)	**<0.001**
Peptic ulcer		9(8.49)	28(10.77)	0.769(0.350–1.690)	0.513
Tumor		18(16.98)	28(10.77)	1.695(0.893–3.217)	0.107
Infection		54(50.94)	56(21.54)	3.783(2.336–6.127)	**<0.001**
Connective tissue diseases		6(5.66)	3(1.15)	5.140(1.261–20.949)	**0.022**
Charlson score	0–1	14(13.21)	129(49.62)		**<0.001**
	2–5	48(45.28)	85(32.69)	5.203(2.702–10.020)	**<0.001**
	≥6	44(41.51)	46(17.69)	8.814(4.424–17.558)	**<0.001**
**Therapeutic process during hospital stay**					
Emergency	Ward 1	9(8.49)	8(3.08)	4.319(1.601–11.652)	**0.004**
	Ward 2	21(19.81)	6(2.31)	13.435(5.199–34.718)	**<0.001**
	Ward 3	3(2.83)	5(1.92)	2.303(0.536–9.902)	0.262
	ICU	11(10.38)	3(1.15)	14.075(3.810–52.000)	**<0.001**
Hospital stay (≥10 days)		90(84.91)	196(75.38)	1.837 (1.006–3.353)	**0.048**
ICU admission		2(1.89)	17(6.54)	0.275(0.062–1.211)	0.088
Blood transfusion		14(13.21)	20(7.69)	1.826(0.885–3.767)	0.103
Enteroscopy		20(18.87)	121(46.54)	0.267(0.155–0.460)	**<0.001**
**Medical history**					
Previous exposure to antibiotics		54(50.94)	79(30.38)	2.379(1.497–3.782)	**<0.001**
Antibiotics exposure during hospital stay	1–2 types	40(37.74)	110(42.31)	3.152(1.257–7.902)	**0.014**
	≥3 types	60(56.60)	98(37.69)	5.306(2.149–13.104)	**<0.001**
Cephalosporins	2^nd^ gen	7(6.60)	16(6.15)	1.474(0.572–3.801)	0.645
	3^rd^ gen	7(6.60)	9(3.46)	2.621(0.925–7.423)	0.070
	4^th^ gen	38(35.85)	56(21.54)	2.286(1.350–3.874)	**0.001**
	≥2 types	8(7.55)	24(9.23)	1.123(0.473–2.668)	0.792
Carbapenems		49(46.23)	61(23.46)	2.804(1.740–4.521)	**<0.001**
Fluoroquinolones	1 type	61(57.55)	104(40.00)	2.542(1.552–4.162)	**<0.001**
	≥2 types	12(11.32)	13(5.00)	4.000(1.674–9.559)	**0.002**
Vancomycin		34(32.08)	71(27.31)	1.257(0.770–2.053)	0.361
Metronidazole		11(10.38)	77(29.62)	0.275(0.140–0.542)	**<0.001**
Cephamycins		11(10.38)	48(18.46)	0.511(0.254–1.028)	0.060
Enzyme inhibitors		20(18.87)	33(12.69)	1.600(0.871–2.939)	0.130
Aminoglycans		10(9.43)	12(4.62)	2.153(0.900–5.147)	0.085
Rifaximin		5(4.72)	23(8.85)	0.510(0.189–1.379)	0.185
PPIs		67(63.21)	153(58.85)	1.201(0.754–1.914)	0.440
Prednisolone		50(47.17)	80(30.77)	2.009(1.264–3.193)	**0.003**
Immune inhibitors		6(5.66)	43(16.54)	0.303(0.125–0.735)	**0.008**

*p-values < 0.05 are indicated in bold type.

As for the non-CDI control group, 106 cases were enrolled and analyzed to reveal the potential risks of infection by the epidemic clone (**Supplementary Table 3**). The average age (49.75 ± 18.35) and hospitalization days(13.98 ± 13.15) of the non-CDI group were significantly lower than the ST81-infected (**Figure 6B**). Of interest, while patients of emergency department were more likely to be infected by ST81 clone, most cases (n=78, 73.58%) of the non-CDI group were from gastroenterology department.

### *Clostridium difficile* Infection Outcome Analysis

**Figure 7:**
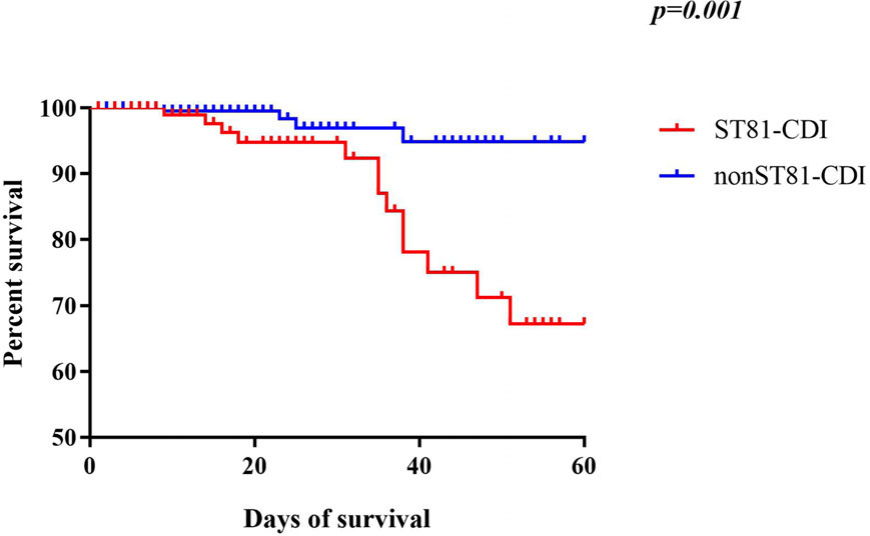
Kaplan-Meier survival curves of patients with ST81 clone infection compared with non-ST81 clone infection (p=0.001 by log-rank test).

**Figure 8:**
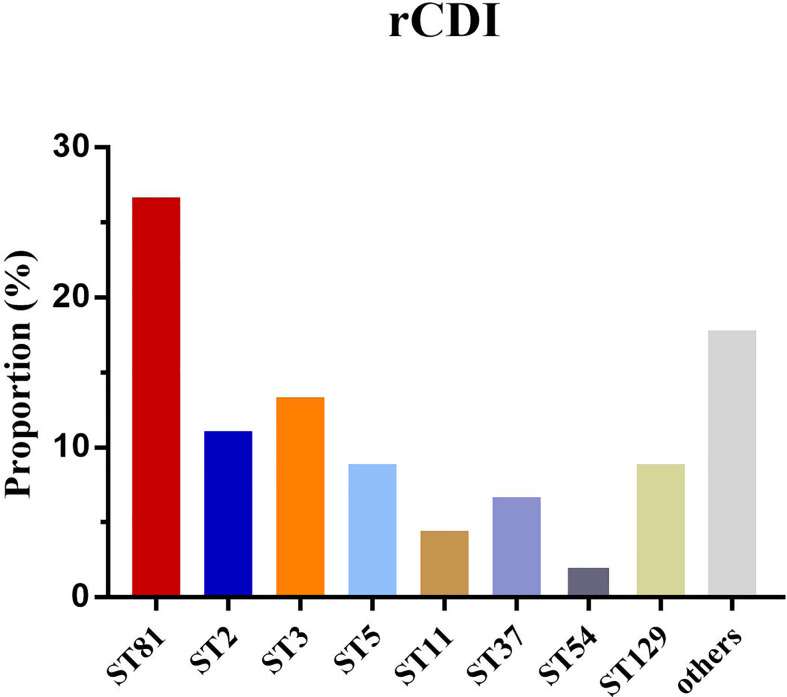
Proportion of major sequence types isolated from recurrent CDI patients.

The mortality of CDI in the ST81-infected (n=12, 11.32%) and non-ST81 CDI (n=6, p=2.31%) groups varied significantly(p<0.001). Moreover, as shown in **Figure 7**, the Kaplan-Meier survival analysis indicated that the 60-day mortality of patients infected by ST81 *C. difficile* was significantly higher than non-ST81 infection group(p=0.001), while the 30-day mortality was of no statistical difference (**Supplementary Figure 2**). The majority of those 18 inpatients were male (n=14, 77.78%), with an average age of 68.71 ± 15.02 years old, while the female patients were much older with a mean age of 83.25 ± 7.46 years old. We further investigated the population with recurrent CDI (See **Figure 8**). Of the 45 strains isolated from the 21 patients, ST81 was also the dominant genotype (n=12, 26.67%), followed by ST3 (n=6, 13.33%), and ST2 (n=5, 11.11%). The proportions of ST5 and ST129 were the same (n=4, 8.89%), and other genotypes accounted for less than 7% (n ≤ 3).

## Discussion

Since the early 2000s, CDI has been an emerging health problem associated with antibiotics abuse reported mainly in North America and Europe (Loo et al., 2005; Kuijper et al., 2006; Jump et al., 2018). While the pooled rate of hospital-acquired CDI was 7.4 cases per 10,000 patient-days (McDonald et al., 2018), the incidence of inpatients was 7.06 cases per 10,000 patient-days in this 5-year study. Thorough and continuous surveillance of CDI has remained inadequate in Asia until the 2010s (Collins et al., 2013). In this retrospective study, we collected all *C. difficile* strains isolated in a general teaching hospital in Shanghai, China over a period of 5 years from September 2014 to August 2019 to investigate the molecular epidemiology and risk factors for the most prevalent genotype infection. Unlike the previously reported predominant clones of *C. difficile* isolates elsewhere in China, which were ST37, followed by ST54, ST3, ST2, and ST35 (Huang et al., 2009; Hawkey et al., 2013; Luo et al., 2019), we observed that ST81 was the major ST type in Renji Hospital in Shanghai, China for these 5 years, indicating a geographical diversity of molecular typing. Notably, a growing trend of ST81 clone in Beijing has been reported recently (Cheng et al., 2020), while another study on *C. difficile* colonization in patients admitted to an intensive care unit in Shanghai demonstrated a predominant role of ST81 genotype (Cui et al., 2019). Given that ST81 strain is becoming epidemic while relevant data is limited, it is of great importance to analyze the risk factors and clinical outcome of ST81 clone infection, as well as to monitor the antimicrobial susceptibility patterns since ST81 has a higher resistance rate to fluroquinolones and clindamycin as previously discovered (Wang et al., 2018b) and has also been demonstrated by this study. Taken together, combined molecular epidemiology and antimicrobial susceptibility analyses of clinical *C. difficile* isolates, as well as analysis of mortality and risk factors for ST81 clone infection, to inform the implementation of preventive management and antibiotic stewardship of *C. difficile* infection clinically.

We divided the 562 isolates into three groups by year (2014–2015, 2016–2017, 2018–2019), containing 120, 193, and 249 isolates respectively, which balanced the proportion of each group to reduce the impact of small sample size. In the current study, a decrease in the prevalence of ST37 and ST129 was observed, accompanied by an increase of ST39. It has been estimated that ST1/RT027, characterized as a hypervirulent clone with excess toxin yield (TcdA+TcdB+CDT+), contributed greatly to the epidemic of CDI and economic burden in western countries (Loo et al., 2005; Guh et al., 2020), while the isolation rate of ST1 in China is relatively low and has first been found in Guangdong, China in 2012 (Jin et al., 2017; Qin et al., 2017), and sporadically reported in the Asia-Pacific region (Wang et al., 2014; Luo et al., 2019). Eight ST1 (1.42%) were isolated from different patients, five of which were admitted into the cadre wards in 2016, implying a potential nosocomial infection due to its higher transmissibility defined as profuse shedding and effective persistence in the environment (Kong et al., 2019). ST81 and ST54 remained the two most prevalent strains in this hospital, while ST81 was found mainly in the emergency department and ST54 tended to be the dominant clone in the gastroenterology department. Different distribution properties of the clones may correlate with specific susceptible population, medical staff, as well as environmental characteristics, of which the intrinsic relationship needs to be further investigated. Unlike the other reports, *tcdA+tcdB+cdt-* strains accounted for most in our study, followed by the *tcdA-tcdB+cdt-* genotype, which appeared to be more prevalent in East Asia according to analyses from mainland China (Xu et al., 2017).

Antibiotics play a Vital role in both eliciting and curing CDI. In this study, the antimicrobial resistance pattern was similar to that which has been previously reported, where all strains were susceptible to vancomycin, teicoplanin, and daptomycin (Collins et al., 2013; Jin et al., 2017; Banawas, 2018; Luo et al., 2019; Thorpe et al., 2019). As one of the first-line medications against *C. difficile* infection, metronidazole was found effective in eradicating *C. difficile* in most cases, while a certain study (Jin et al., 2017) has reported an unusual 15.6% high-level resistance rate to metronidazole of which the potential mechanisms are under investigation. Six isolates (1.07%) were found resistant to metronidazole in our study, however, the MIC_50_ and MIC_90_ of metronidazole were 0.125 and 0.25 mg/L, respectively, with a continuous decreasing MIC value in this 5-year period, consistent with that discovered by Thorpe et al. from 2013 to 2016 (Thorpe et al., 2019). This diminishing trend of MIC value may be related to the withdrawal of metronidazole from first-line therapy for uncomplicated CDI (McDonald et al., 2018). Another antibiotic frequently used and showing a decreasing MIC value was meropenem, with the resistance rate changing from 14.41% to 5.28% in this 5-year period, while some studies in Thailand and China reported no resistant strain to meropenem (Putsathit et al., 2017; Wang et al., 2018a). As has been found in the Asia-Pacific region and western countries (Luo et al., 2019; Thorpe et al., 2019), high resistance rate to clindamycin, ranging from 60.53% to 76.68% was also verified in our study. Although the proportion of clindamycin resistant *C. difficile* isolates remained around 76% between 2016 and 2019, the MIC value increased significantly, indicating a potential rise in the resistance rate. Moxifloxacin belongs to the class of fluoroquinolones, which are broad-spectrum antibiotics and considered as a predominant risk factor for *C. difficile-*associated diarrhea (Pépin et al., 2005). We found that the resistance rate to moxifloxacin in this study remained stable in these years (43.29%), while ST81 showed significantly higher resistance rate and MIC_50_ than all the other STs. A multi-center study in Beijing, China has revealed that amino acid mutations in the *gyrA* and *gyrB* genes primarily underlies the resistance mechanism of fluoroquinolones (Cheng et al., 2020).

Our study additionally compared demographics, underlying diseases, and therapeutic process during hospitalization and usage of medication between ST81 CDI and the two control groups by univariate regression model and then included the variables significantly different (p<0.05) between the case and control groups in a multivariate analysis. In accordance with previous studies, longer hospital stay and more complications are strong indicators of CDI (Qin et al., 2017; Jump et al., 2018; Lee et al., 2019), while the age threshold as a risk factor for all CDI patients was 55 years in China (Jin et al., 2017) and 65 years in Europe (Czepiel et al., 2019). Our study is supportive of the view that PPIs use may increase the chance of CDI, while it is still controversial and lacks sufficient evidence for discontinuation of PPIs for prevention (McDonald et al., 2018). Although the univariate analyses revealed that Charlson score was higher in the ST81 CDI group, which tended to have diseases including hypertension, diabetes mellitus, chronic kidney diseases, chronic liver diseases, connective tissue diseases, and cardio-cerebrovascular diseases, after integrating various factors by the multi-variable analyses, only suffering from chronic kidney diseases and connective tissue diseases increased the risk of ST81 CDI 2 to 7-fold and 2 to 53-fold, respectively. A previous study in Korea has also indicated that advanced chronic kidney disease is a strong risk factor for CDI, which may be correlated with systemic chronic inflammation and subsequent immunosuppression that leads to elevated susceptibility to CDI (Kim et al., 2016). Only one study has found a significant association between connective tissue diseases and 30-day mortality of CDI (Xu et al., 2017), while patients with connective tissue diseases in our study were more prone to ST81 CDI. Since the ST81 CDI group appeared to be burdened with more inflammation caused by renal diseases and/or connective tissue diseases and represented severe clinical characteristics, prednisolone was more frequently applied to these patients. Furthermore, antibiotics exposure before and during hospitalization has been closely related to CDI either discovered in this study or in others (Alicino et al., 2016; Xu et al., 2017), suggesting that disturbance of gut microbiota by antibiotics may lead to the proliferation of resistant *C. difficile* strains and then infection by toxigenic clones. With respect to antibiotics exposure in hospital, the usage of cephalosporin, fluoroquinolones, carbapenems, and metronidazole were found to be significantly different between the case and control groups. While previous analyses have pointed out exposure to cephalosporins (2^nd^, 3^rd^, and 4^th^ generation), clindamycin, fluoroquinolones, carbapenems, penicillins as well as trimethoprim/sulfonamides were associated with an increased risk of hospital-acquired CDI (Slimings and Riley, 2013), it did not contribute to an inclination for ST81 clone infection specifically. Of note, usage of metronidazole appeared to be a protective factor for ST81 CDI (OR: 0.196, CI: 0.072–0.535 p=0.001). Only 11 out of 106 patients infected with ST81 clone were prescribed metronidazole and all ST81 isolates from inpatients were susceptible to it, which may explain the effectiveness of metronidazole therapy even though it is currently no longer recommended as regular medication. Additionally, longer hospital stay increased the risk of infection by ST81 clone (OR:3.232,CI: 1.410–7.409 p=0.006). More importantly, since inpatients infected with ST81 clone were mainly from the emergency department, we further analyzed whether certain wards of that department were a risk factor for ST81 infection. According to the multivariate analyses, admission to emergency ward 2 and emergency ICU significantly increased the chance of ST81 infection (OR: 5.954 and 32.803 respectively). As previously described, the ST81 clone was responsible for a nosocomial patient-environment-patient transmission in the emergency department in 2015, so we suspect that part of the ST81 clone survived the sterilization measures and continuously lurked in the hospital environment as spores, which should be further analyzed by sequencing to explore the phylogenetic relationships and gene editing tools to verify specific genes contributing to the high persistence and transmissibility of spores from ST81 clone.

In addition, we further investigated the outcome (60-day all-cause mortality and recurrence) of the first case and control groups. Genotype ST81 was correlated with a higher mortality compared with the other STs, while it was noted in another study that CDT+ clones or ST-5 infected patients were predisposed to higher mortality (Xu et al., 2017). However, as an acute disease, ST81 infection was not associated with a higher 30-day mortality (**Supplementary Figure 2**), but contributed to a chronic inflammation which aggravated the development of the underlying disease as indicated by the increased 60-day mortality. ST81 CDI associated mortality may partly be attributed to the lack of awareness of *C. difficile* associated diarrhea (CDAD) clinically, leading to inappropriate treatment, since ST81 infected patients had more complications with sophisticated clinical manifestations. Moreover, the ST81 clone has less toxin production than non-ST81 genotypes (Qin et al., 2017), resulting in a lower positive rate of toxin detection clinically, which misled the therapeutic regime and allowed the pathogen to persist longer in patients, causing further damage in a prolonged period. The ST81 clone accounted for most of the recurrent CDI cases, possibly due to a low virulence and high multidrug resistance rate, and also correlated with a susceptible population and persistence in various environments. Notably, the appearance of ST5 and ST11, two hypervirulent genotypes with all toxin genes positive (*tcdA+tcdB+CDT+*) and accounting for 8.89% and 4.44% respectively, should arouse attention for close monitoring since they were prevalent in western countries and may cause severe clinical presentations.

In summary, *C. difficile* ST81 was found to be a dominant genotype epidemic in this general teaching hospital, with higher resistance rates to multiple antibiotics including moxifloxacin, meropenem, clindamycin and rifaximin. Emergency ward 2 and emergency ICU were high-risk ST81 clone outbreak departments in this hospital, thus routine surveillance and monitoring of *C. difficile* spores should be initiated. If diarrhea occurs in patients with chronic kidney diseases or connective tissues diseases, management of diarrhea should follow the CDI treatment guidelines. Long-term and elderly inpatients are also high-risk populations in our study. Although the major clone reported has been ST37 in China, ST81 is genetically close to ST37 and has higher sporulation ability and less toxin production, which may contribute to its persistence in the external environment and colonization in the gut (Wang et al., 2018b; Cui et al., 2019). Some local epidemics of ST81 clone have already been noted in northern and eastern China, so it is of vital importance to initiate continuous surveillance for CDI, as well as raise awareness of physicians to follow appropriate antibiotic stewardship of CDI in the first place, which is essential in preventing unnecessary mortality.

## Data Availability Statement

The original contributions presented in the study are included in the article/**Supplementary Material**, further inquiries can be directed to the corresponding author.

## Ethics Statement

This study was approved by The Institutional Review Board of the Renji Hospital, School of Medicine, Shanghai Jiaotong University, Shanghai, China. No consent was needed for this study.

## Author Contributions

ZY, QH, JQ, and ML contributed to the conception and design of the study. QH and JQ organized the database. ZY performed the statistical analysis. ZY and QH plotted the figures and tables in this work. ZY and QH wrote the first draft of the manuscript. ZY, QH, JQ, and ML wrote sections of the manuscript. All authors contributed to the article and approved the submitted version.

## Funding

This work was supported by the innovative research team of high-level local universities in Shanghai, the National Natural Science Foundation of China (grant numbers 81873957, 81861138043, 81772139, 81902118), project of Shanghai Science and Technology Commission (19JC1413005), and Excellent talents training plan of Shanghai public health system (GWV-10.2-YQ08).

## Conflict of Interest

The authors declare that the research was conducted in the absence of any commercial or financial relationships that could be construed as a potential conflict of interest.
