# Supplementary material for: Molecular Epidemiology and Risk Factors of Clostridium difficile ST81 Infection in a Teaching Hospital in Eastern China
Source: Front Cell Infect Microbiol. 2020 Dec 23;10:578098. doi: 10.3389/fcimb.2020.578098 (PMC7785937; doi:10.3389/fcimb.2020.578098)
Supplement: Supplementary file 1 [file DataSheet_1.docx]

**Supplementary Materials**

Supplementary Figures


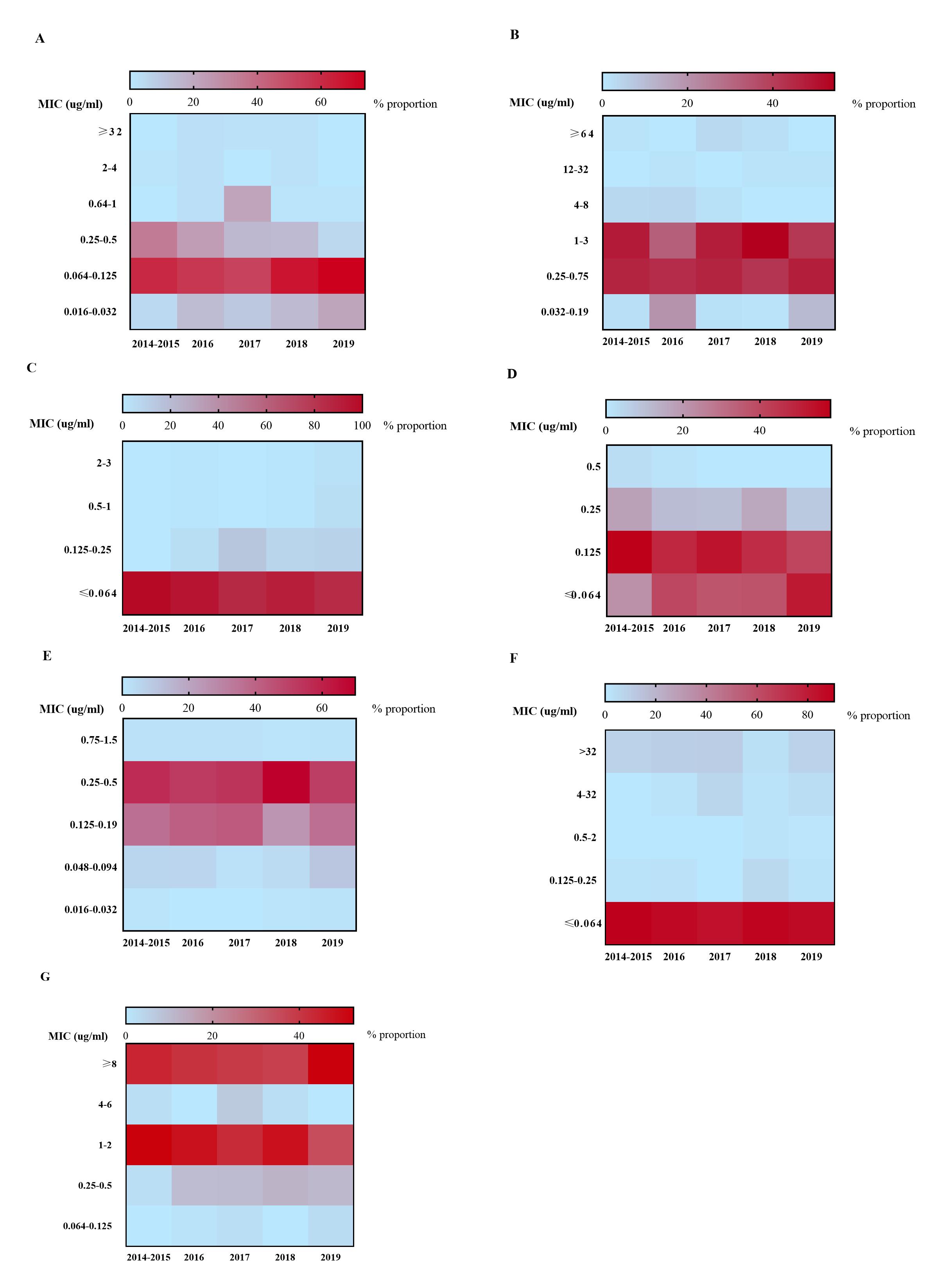


**Supplementary Figure 1.** MIC distribution of metronidazole (A), linezolid (B), daptomycin (C), teicoplanin (D), vancomycin (E), rifaximin (F) and moxifloxacin (G).


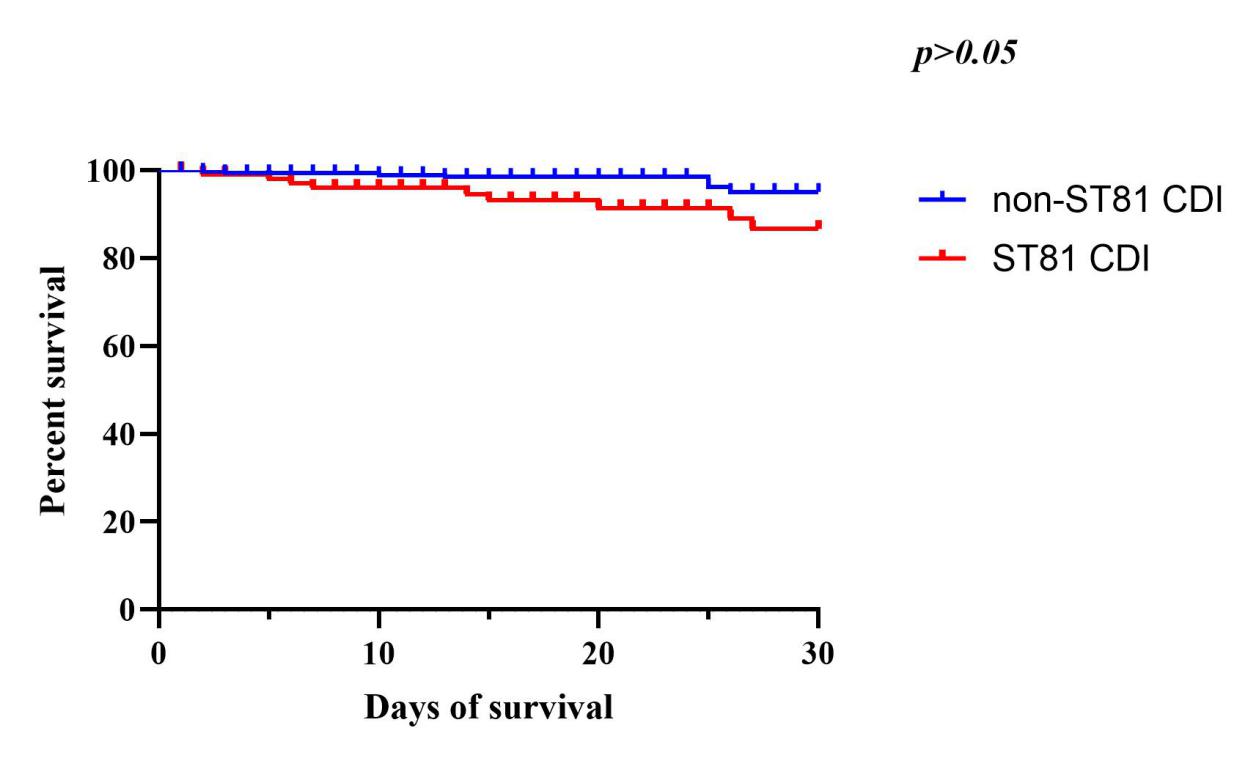


**Supplementary figure 2**. All-cause 30-day mortality of the case and control groups from 2014 to 2019.

**Supplementary Table 1**. Primers used for molecular typing.

| Gene | Primer | Sequencing(5’-3’) |
| --- | --- | --- |
| *adk* | adk-F | TTACTTGGACCTCCAGGTGC |
|  | adk-R | TTTCCACTTCCTAAAGGCTGC |
| *atpA* | atpA-F | TGATGATTTAAGTAAACAAGCTG |
|  | atpA-R | AATCATGAGTGAAGTCTTCTCC |
| *dxr* | dxr-F | GCTACTTTCCATTCTATCTG |
|  | dxr-R | CCAACTCTTTGTGCTATAAA |
| *glyA* | glyA-F | ATAGCTGATGAGGTTGGAGC |
|  | glyA-R | TTCTAGCCTTAGATTCTTCATC |
| *recA* | recA-F | CAGTAATGAAATTGGGAGAAGC |
|  | recA-R | ATTCAGCTTGCTTAAATGGTG |
| *sodA* | sodA-F | CCAGTTGTCAATGTATTCATTTC |
|  | sodA-R | ATAACTTCATTTGCTTTTACACC |
| *tpiA* | tpiA-F | ATGAGAAAACCTATAATTGCAG |
|  | tpiA-R | TTGAAGGTTTAACTTCCACC |
| *tcdA* | tcdA-F | AGATTCCTATATTTACATGACAATAT |
|  | tcdA-R | GTATCAGGCATAAAGTAATATACTTT |
| *tcdB* | tcdB-F | GTGTAGCAATGAAAGTCCAAGTTTACGC |
|  | tcdB-R | CACTTAGCTCTTTGATTGCTGCACCT |
| *cdtA* | cdtA-F | TGAACCTGGAAAAGGTGATG |
|  | cdtA-R | AGGATTATTTACTGGACCATTTG |
| *cdtB* | cdtB-F | CTTAATGCAAGTAAATACTGAG |
|  | cdtB-R | AACGGATCTCTTGCTTCAGTC |

**Supplementary Table 2**. Characteristics of the ST81-infected and non-CDI groups.

| Variables | | ST81 CDI(n=106) | Non-CDI  (n=106) | Univariate analysis | |
| --- | --- | --- | --- | --- | --- |
|  |  | N (%) | N (%) | OR (95% CI) | p-value |
| Demographics | |  |  |  |  |
| Age (years, x±SD) | | 64.73±17.68 | 49.75±18.35 | 1.046(1.028-1.063) | <0.001 |
| Gender: male | | 68(64.15) | 68(64.15) |  |  |
| Diseases | |  |  |  |  |
| Hypertension | | 41(38.68) | 13(12.26) | 4.512(2.241-9.084) | <0.001 |
| Diabetes mellitus | | 22(20.75) | 7(6.60) | 3.289(1.327-8.154) | 0.01 |
| Chronic kidney diseases | | 46(43.40) | 11(10.38) | 6.621(3.181-13.78) | <0.001 |
| Chronic liver diseases | | 34(32.08) | 21(19.81) | 1.911(1.02-3.582) | 0.043 |
| Cardio-cerebrovascular diseases | | 47(44.34) | 14(13.21) | 5.235(2.651-10.337) | <0.001 |
| Peptic ulcer | | 9(8.49) | 8(7.55) | 1.137(0.421-3.068) | 0.8 |
| Tumor | | 18(16.98) | 9(8.49) | 2.205(0.942-5.161) | 0.069 |
| Infection | | 54(50.94) | 15(14.15) | 6.300(3.237-12.260) | <0.001 |
| Connective tissue diseases | | 6(5.66) | 3(2.83) | 2.06(0.501-8.463) | 0.316 |
| Charlson score(x±SD) | | 4.45±2.59 | 2.010±2.045 | 1.572(1.357-1.822) | <0.001 |
| Therapeutic process during hospital stay | |  |  |  |  |
| Department | GD^a^ | 28(26.42) | 78(73.58) | 0.169(0.081-0.352) | <0.001 |
|  | ED^b^ | 44(41.51) | 12(11.32) | 1.725(0.721-4.127) | 0.22 |
| Hospitalization  (days,x±SD) | | 29.96±30.91 | 13.98±13.15 | 1.06(1.036-1.086) | <0.001 |
| Blood transfusion | | 14(13.21) | 5(4.72) | 3.074(1.066-8.868) | 0.038 |
| Enteroscopy | | 20(18.87) | 73(68.87) | 0.105(0.056-0.199) | <0.001 |
| Medical history | |  |  |  |  |
| Cephalosporins | | 61(57.55) | 33(31.13) | 2.999(1.707-5.267) | <0.001 |
| Carbapenems | | 49(46.23) | 3(2.83) | 29.515(8.803-98.955) | <0.001 |
| Fluoroquinolones | | 72(67.92) | 23(21.70) | 7.642(4.127-14.151) | <0.001 |
| Vancomycin | | 34(32.08) | 6(5.66) | 7.87(3.139-19.734) | <0.001 |
| Metronidazole | | 11(10.38) | 11(10.38) | 1.103(0.464-2.622) | 0.825 |
| Rifaximin | | 5(4.72) | 10(9.43) | 0.475(0.157-1.441) | 0.189 |
| PPIs | | 67(63.21) | 32(30.19) | 3.973(2.241-7.043) | <0.001 |
| Prednisolone | | 50(47.17) | 17(16.04) | 4.674(2.455-8.899) | <0.001 |
| Immune inhibitors | | 6(5.66) | 4(3.77) | 1.530(0.419-5.585) | 0.52 |

^a^GD: Gastroenterology Department.

^b^ED: Emergency Department.
